# Green Polymer Chemistry: Enzyme Catalysis for Polymer Functionalization

**DOI:** 10.3390/molecules20059358

**Published:** 2015-05-21

**Authors:** Sanghamitra Sen, Judit E. Puskas

**Affiliations:** Department of Chemical and Biomolecular Engineering, The University of Akron, Akron, OH 44325, USA; E-Mail: ssen@uakron.edu

**Keywords:** enzyme catalysis, *Candida antarctica* lipase B, transesterification, Michael addition, polymer functionalization, poly(ethylene glycol), polyisobutylene, polysiloxanes, polystyrene, regioselectivity, chemoselectivity

## Abstract

Enzyme catalyzed reactions are green alternative approaches to functionalize polymers compared to conventional methods. This technique is especially advantageous due to the high selectivity, high efficiency, milder reaction conditions, and recyclability of enzymes. Selected reactions can be conducted under solventless conditions without the application of metal catalysts. Hence this process is becoming more recognized in the arena of biomedical applications, as the toxicity created by solvents and metal catalyst residues can be completely avoided. In this review we will discuss fundamental aspects of chemical reactions biocatalyzed by *Candida antarctica* lipase B, and their application to create new functionalized polymers, including the regio- and chemoselectivity of the reactions.

## 1. Introduction

“Green chemistry” is still an emerging field that strives to work at the molecular level to achieve sustainability. The field has received widespread interest in the past decade due to its ability to harness chemical innovation to simultaneously meet environmental and economic goals [1]. Synthesis of polymeric materials with unique properties can be achieved not only by polymerization reactions but also by modification/functionalization of existing polymers. Enzymatic catalysis is an alternative strategy to increase the diversity of functional groups in polymeric materials [2,3,4,5]. Specifically, enzyme-catalyzed polymer functionalization carried out under solventless conditions is a great advancement in the design of green processes for biomedical applications, where the toxicity of solvents and catalyst residues needs to be considered. The polymers obtained through this method are free from any metal catalyst, which often broadens the application of the products, particularly for biomedical use. Our group pioneered enzyme-catalyzed quantitative polymer functionalization that will be discussed below in more detail.

Enzymes are Nature’s catalysts that accelerate specific metabolic reactions in living cells. As an environmentally friendly alternative to conventional chemical catalysts, enzymes offer several advantages, including high selectivity, high efficiency, the ability to operate under mild conditions and catalyst recyclability. Answers to the question “Why use enzymes?” include necessity, convenience and opportunity. New synthetic and catalytic methods are necessary to design new classes of functional polymers for covalent polymer-drug conjugates. Despite all the advantages that enzymatic catalysis offers, the area of quantitative functionalization of preformed polymers has not fully been developed. Enzymes can catalyze the modification of a polymer through functional groups located in the main chain, in the side chains or at the polymer terminals (synthetic symmetric or asymmetric telechelic polymers). 

**Figure 1:**
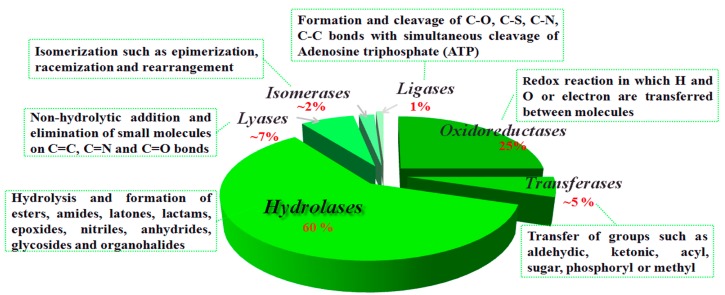
Classification and function of enzymes. Reprinted with permission from [6]. Copyright © 2011 Elsevier.

Enzymes are classified into six main groups according to the International Union of Biochemistry and Molecular Biology (Figure 1) [6]. Today about 3000 enzymes are available commercially and some of them are mutated for industrial applications. Generally, oxidoreductases, hydrolases, and isomerases are relatively stable, and the most widely used catalysts in biotransformations. Among these, some of the isolated enzymes are conveniently used as catalysts in practice. In contrast, lyases and ligases are present in lesser amounts in living cells and are less stable for isolation or separation from living organisms.

Lipases catalyze the hydrolysis of triglyceride substrates into fatty acids and glycerol at the lipid-water interface *in vivo.* They are widely used in esterification, transesterification, aminolysis, and Michael addition reactions in organic solvents [7]. These enzymes display almost no activity as long as the substrate is in a monomeric state. However, when the substrate concentration is beyond its critical micelle concentration, a lipophilic phase forms and a sharp increase in lipase activity takes place. This phenomenon is called “interfacial activation” [8,9]. Interfacial activation is attributed to a rearrangement process within the enzyme *in vitro* [8,10]. X-ray structural analysis of lipases usually shows a “closed” conformation where a “lid” blocks the active site. This lid opens when the enzyme is exposed to an interface of a biphasic water-oil system and thus the catalytic activity of the lipase increases [11]. Lipases also work in organic solvents. The most useful lipases for organic synthesis are: *Porcine pancreatic lipase* (PPL), lipase from *Pseudomoanas cepacia* (Amano lipase PS), lipase from *Candida rugosa* (CRL), and lipase B from *Candida antarctica* (CALB) [12]. 

Candida antarctica Lipase B (CALB) 

**Figure 2:**
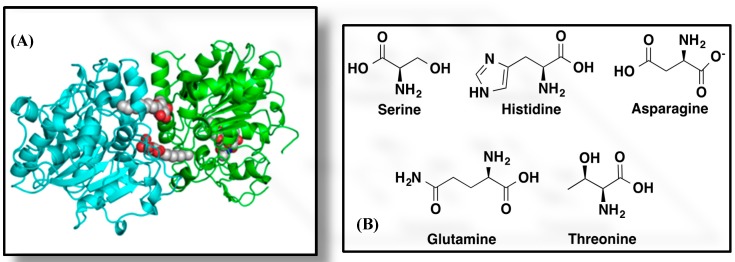
(**A**) 3D structure of CALB [16]; X-ray-diffraction with resolution of 2.10 Å created with Polyview 3D; (**B**) Structures of the amino acid residues forming the catalytic triad and the oxyanion hole of CALB.

The structure of CALB was solved in 1994 and is shown in Figure 2A [13]. This enzyme belongs to the α/β-hydrolase-fold superfamily [14], which contains enzymes that have evolved from a common ancestor to catalyze reactions as various as hydrolysis of esters, thioesters, peptides, epoxides, and alkyl halides or cleavage of carbon bonds in hydroxynitriles [12]. CALB is made of 317 amino acids and has a molecular weight of 33 kDa [13]. The active site pocket of CALB, which is approximately 10Å x 4Å wide and 12 Å deep [13], is illustrated in Figure 2A. It contains the catalytic triad, Ser105-His224-Asp187 (Figure 2B), common to all serine hydrolases [15,16,17]. The top pocket is the “carbonyl” pocket an oxyanion hole that stabilizes the transition state and the oxyanion in the reaction intermediate. This oxyanion hole is a spatial arrangement of three hydrogen-bond donors, one from the side chain of Thr40 and two from the backbone amides of Thr40 and Gln106. The bottom is the “hydroxyl” pocket.

In most reported biotransformations CALB is used physically immobilized on a macroporous poly(methyl methacrylate) resin and is commercially available as Novozyme 435. The resin beads have diameters in the range of 0.3–0.9 mm, with 20 wt % protein and 1–2 wt % water content bound around the protein [18].

As a catalyst for polymerization, CALB has successfully been applied over the last two decades in two major polymerization strategies, namely ring-opening polymerization (ROP) of lactones and polycondensation-type reactions [19,20,21]. Moreover, important chemical reactions, specifically, transesterification, Michael addition and epoxidation can be performed under much milder conditions exploiting the catalytic activity of CALB. Here we will concentrate on CALB-assisted functionalization of polymers with emphasis on region- and chemoselectivity controlled through the use of the enzyme, with first discussing some fundamental aspects established in small molecule reactions. 

## 2. Fundamental Aspects of CALB-Catalyzed Transesterification, Michael Addition and Epoxidation 

As mentioned above, due to high efficiency, recyclability, ability to react under milder conditions, and easy separation, immobilized CALB-assisted functionalization of polymers are of great importance. In this section we will discuss CALB-catalyzed transesterification, Michael addition and epoxidation reactions that have been used for the functionalization of polymers. First we will discuss some fundamental aspects of enzyme-catalyzed transesterification, Michael addition and epoxidation.

### 2.1. CALB-Catalyzed Transesterification

**Scheme 1:**
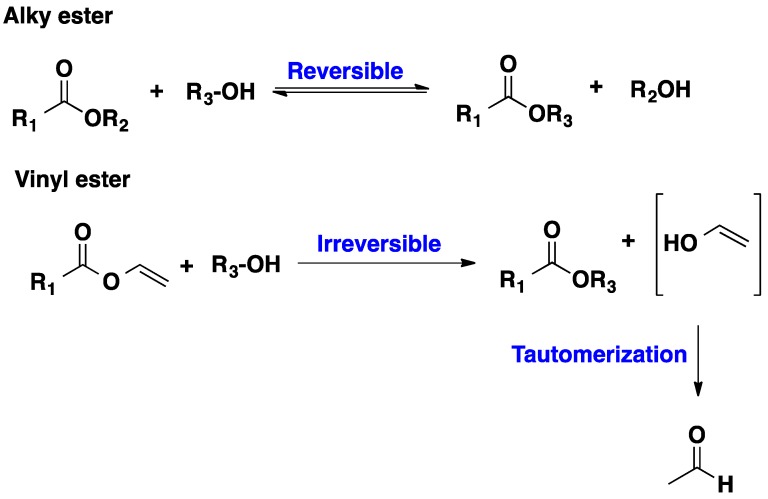
Transesterification of esters with alcohols: Reversible with an alkyl ester or a halogenated alkyl ester, and Irreversible with a vinyl ester [21].

Classical transesterification reactions can be catalyzed by CALB. Transesterification reactions are generally reversible but the equilibrium can be shifted towards the product if the side product is removed or the nucleophilicity of the leaving group of the acyl donor is reduced by the introduction of electron-withdrawing groups (e.g., trihaloesters, enol esters, oxime esters, anhydrides, *etc.*) [22]. The use of enol esters [23] such as vinyl or isopropenyl esters appears to be the most useful since they liberate unstable enols as by-products, which rapidly tautomerize to give the corresponding aldehydes or ketones (Scheme 1). Therefore, the reaction becomes completely irreversible. 

Yadav *et al.* compared the catalytic activity of various commercially available lipases in transesterification of vinyl acetate with n-octanol. CALB was found to be the most active catalyst in heptane as a solvent [24]. Acetaldehyde, which forms during the reactions with vinyl esters, is known to inactivate the lipases from *Candida rugosa* and *Geotrichum candidum* by forming a Schiff’s base with the lysine residues of the protein; however most lipases, including CALB, tolerate the liberated acetaldehyde [25]. 

**Figure 3:**
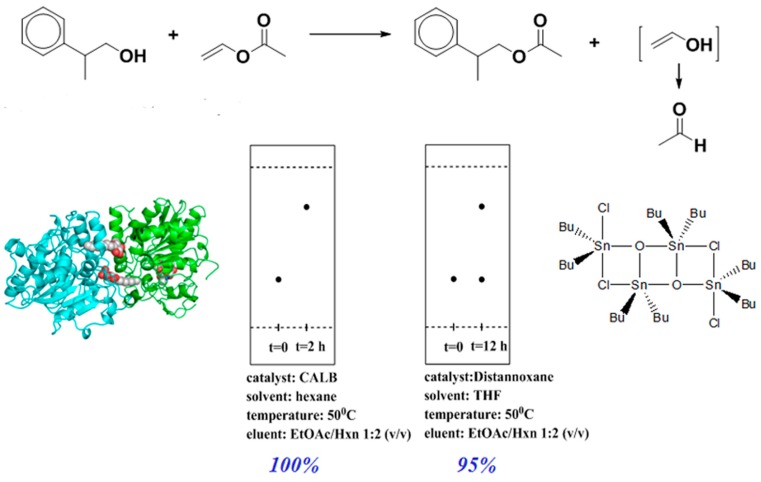
Transesterification of vinyl acetate: comparison of CALB and a tin-based catalyst. Reprinted with permission from [6]. Copyright © 2011 Elsevier.

In a previous report from our group the efficiency of CALB-catalyzed transesterification was demonstrated by comparison with tin octoate; this latter gave 95% conversion when reacting vinyl-acetate with 2-phenylpropane-1-ol in THF in 12 h, while CALB yielded 100% conversion of the vinyl-acetate in hexane in 2 h (Figure 3) [6].

**Figure 4:**
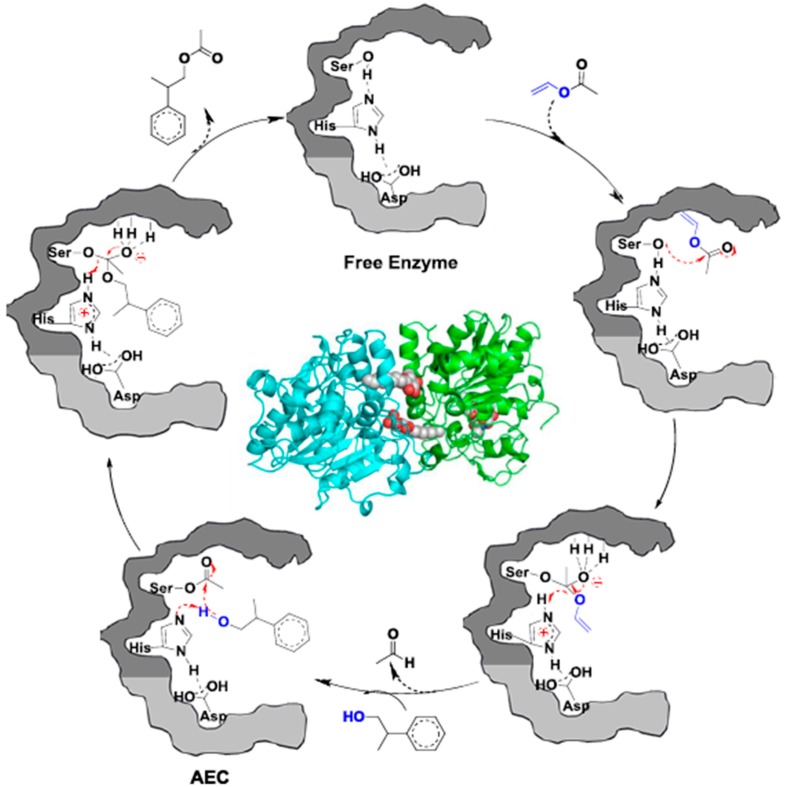
Illustration of the mechanism of CALB-catalyzed transesterification of vinyl acetate with 2-phenylpropane-1-ol. The different shading represents the two enzyme pockets [21].

The catalytic cycle of the CALB-catalyzed transesterification of vinyl acetate (VA) with 2-phenylpropane-1-ol (PPOH) is visualized in Figure 4 based on the mechanism from Bornscheuer and Kazlauskas [12,26]. First, the nucleophilic serine (Ser105) residue interacts with the carbonyl group of the VA, forming a tetrahedral intermediate, which is stabilized by the oxyanion hole of the enzyme via three hydrogen bonds, one from glutamine (Gln106) and two from threonine (Thr40). In the second step vinyl alcohol is released and an acyl-enzyme complex (AEC) is formed. The vinyl alcohol immediately tautomerizes to acetaldehyde making the reaction irreversible. In the third step, the HO– group of PPOH reacts with AEC to form a second tetrahedral intermediate, which is again stabilized by the oxyanion hole. In the last step, the enzyme is deacylated to form the ester product. 

Although an increase in ester concentration was shown to increase the rate and conversion of transesterification [24,27], it was also shown that an increase in alcohol concentration might cause reduced rates and conversions due to competitive inhibition by the alcohol which can bind reversibly to the enzyme active site and prevent the binding of the ester substrate [24,28]. The driving force for alcohol binding might be the high polarity of the region around the active serine site of the enzyme [29].

**Figure 5:**
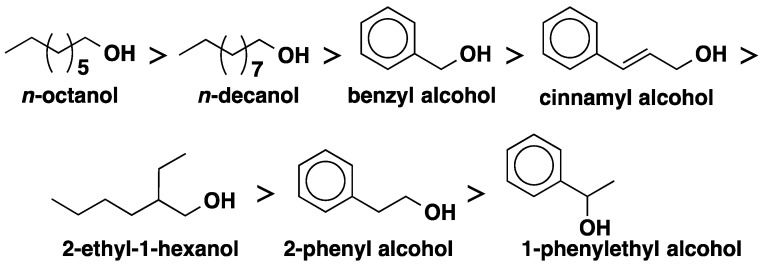
Order of reactivity of various alcohols in CALB-catalyzed transesterification of vinyl acetate.

The structure of the alcohol is also an important parameter affecting the rate and overall conversions in enzymatic transesterification. It was observed that straight-chain alcohols gave better conversion compared to aromatic and branched-chain alcohols in the CALB-catalyzed transesterification of vinyl acetate due to less steric hindrance around the hydroxyl group (Figure 5) [24]. However, longer chains led to slower reaction. Furthermore, aromatic alcohols with saturated shorter side chains (e.g., benzyl alcohol) were more reactive than those with longer but unsaturated side chains (e.g., cinnamyl alcohol). The resonance stability may be playing a role to reduce the reactivity of cinnamyl alcohols.

**Scheme 2:**
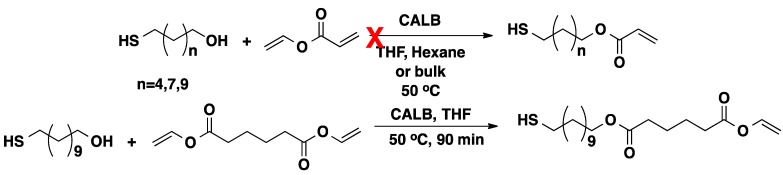
Transesterification of VAcr and DVA with mercapto-alcohols in the presence of CALB.

The chemoselectivity of CALB-catalyzed transesterification was demonstrated with thio-alcohols. α-HO-ω-SH-functionalized molecules have been used in protein modifications [30], gold nanoparticle functionalization [31] and cancer radiotherapy [32] in biomedical applications. The thiol group is very reactive so that chemical protection is often required in the modification of free-SH bearing compounds [33]. The transesterification of vinyl acrylate (VAcr) with mercapto-alcohols (6-mercapto-1-hexanol, 9-mercapto-1-nonanol and 11-mercapto-1- undecanol) was performed using CALB in THF, hexane or in bulk (Scheme 2). No progress of the reaction was observed up to 24 h. However, when VAcr was substituted with divinyl adipate (DVA) the reaction was complete within 90 min [34]. The ^1^H-NMR spectrum shows that the product obtained constitute one thiol end group and one vinyl end group which confirms only one vinyl group of DVA participated in the transesterification reaction [34]. This demonstrates the chemoselectivity of CALB.

**Scheme 3:**
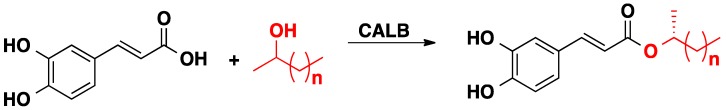
CALB-catalyzed asymmetric synthesis of (*R*)-caffeic acid esters.

Recently Xiao *et al.* have reported the enantioselective esterification of caffeic acid using CALB as shown in Scheme 3. Irrespective of the stereochemistry of the alcohol used, 100% *R* isomer was selectively formed in this esterification reaction when a hydrocarbon, isopropyl ether or 1,4-dioxane was used as the solvent. However, 100% *S* isomer was obtained when THF was used as the solvent [35]. 

### 2.2. CALB-Catalyzed Michael Additions

**Figure 6:**
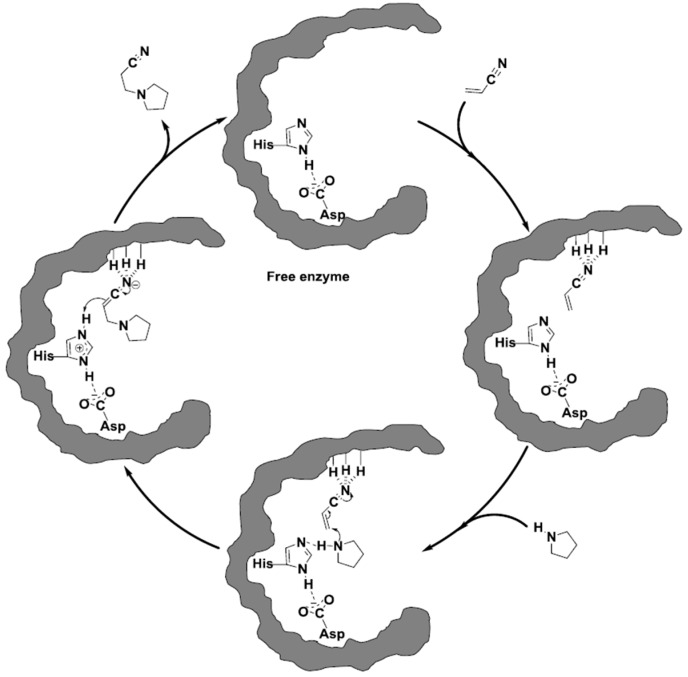
Suggested mechanism for the CALB-catalyzed Michael addition of pyrrolidine to acrylonitrile [21].

The Michael addition, which is a conjugate addition-type reaction, is a powerful and widely used method for the formation of new carbon-carbon and carbon-heteroatom bonds. Michael-type reactions typically involve the use of either strongly basic or acidic conditions, leading to the generation of potentially hazardous waste by-products and/or undesired side products [12]. Bhanage *et al.* reported an efficient enzymatic protocol for the synthesis of β-amino esters via Michael addition of *primary* and *secondary* amines to acrylates using CALB as a biocatalyst [36]. CALB was found to be the most efficient lipase to catalyze the reaction while other lipases led to low yield ranging from 10% to 36% of the desired product. In the absence of CALB under the same reaction conditions only traces of the Michael addition product were obtained [36]. Gotor *et al.* demonstrated CALB-catalyzed Michael-type addition of secondary amines to acrylonitriles and proposed that the serine in the active site was not involved in the reaction [37]. These additions take place when α,β-unsaturated systems are used as the electrophile moiety and amines as the nucleophile substrate. Some evidence of the mechanism of this “promiscuous” activity of CALB points out that the oxyanion hole (Thr40 and Gln106) of the active site stabilizes the negative charge of the transition state while the His224-Asp187 pair facilitates proton transfer during the catalysis. Consequently, it is proposed that solvents of low polarity may induce interaction between the oxyanion hole and the carbonyl oxygen in the catalytic intermediate complex, allowing the ability of CALB to carry out this reaction. According to the suggested mechanism, [37,38,39] the catalytic cycle of the CALB-catalyzed Michael addition of acrylonitrile was visualized as shown in Figure 6 [27,28]. 

First is the interaction with the Michael acceptor, the nitrile group (or carbonyl group in the case of α,β-unsaturated carbonyl compounds) of acrylonitrile is activated by the oxyanion hole of the enzyme. Then the conjugate addition of the incoming nucleophile, *i.e.*, pyrrolidine, to the α-carbon of the Michael-acceptor takes place resulting in an intermediate which is stabilized by both the histidine-aspartate pair and the oxyanion hole in the enzyme active site. In the last step, the histidine-aspartate pair catalyzes the proton transfer from the pyrrolidine to the α-carbon of acrylonitrile [21].

**Scheme 4:**
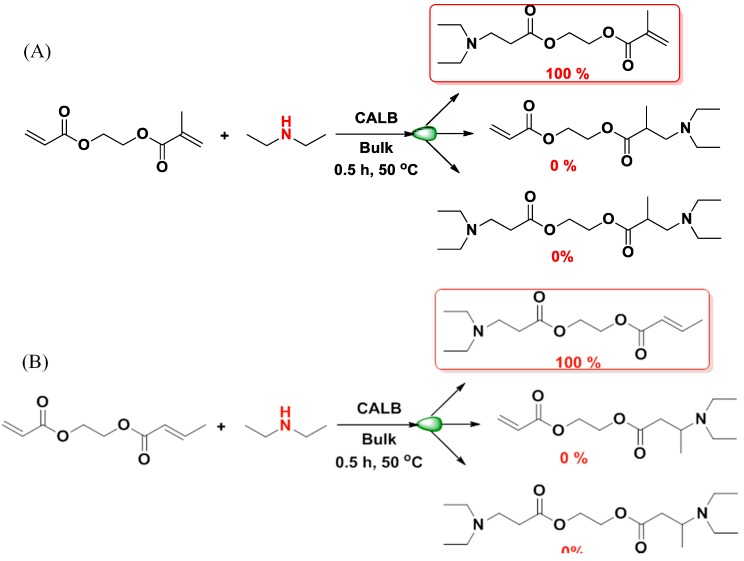
Enzymatic Michael addition of diethylamine to (**A**) α-Acrylated, ω-methacrylated EG in bulk (**B**) α-Acrylated, ω-crotonated EG in bulk [34].

The chemoselectivity/stereospecificity of enzyme-catalyzed Michael addition is demonstrated by the reaction of diethylamine with 2-(acryloyloxy)ethyl acrylate, 2-(acryloyloxy)ethyl methacrylate and 2-(acryloyloxy)ethyl crotonate. The reaction of the acrylate group was complete within 30 min, while the methacrylate and crotonate groups remained intact (Scheme 4). 

**Scheme 5:**
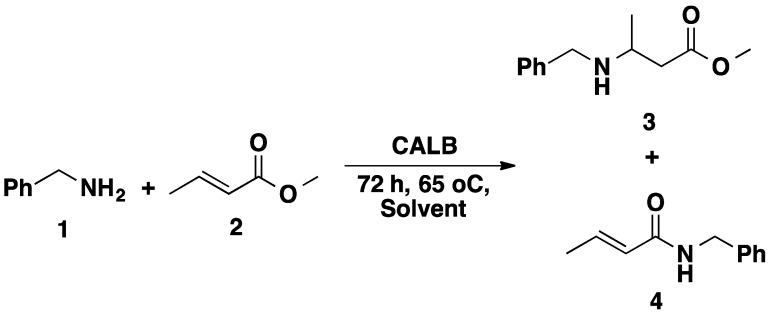
Chemoselectivity of CALB-catalyzed Michael addition.

Castillo *et al.* implemented a “solvent engineering strategy” in order to control the chemoselectivity in a CALB-catalyzed Michael addition reaction of phenylethylamine to methyl crotonate (Scheme 5) [40]. Chemoselectivity of the enzymatic process was elucidated in terms of polarity of the medium: the Michael adduct 3 preferentially accumulated in hydrophobic medium, whereas in polar solvents the amide 4 formed preferentially.

### 2.3. CALB-Catalyzed Epoxidation 

**Scheme 6:**
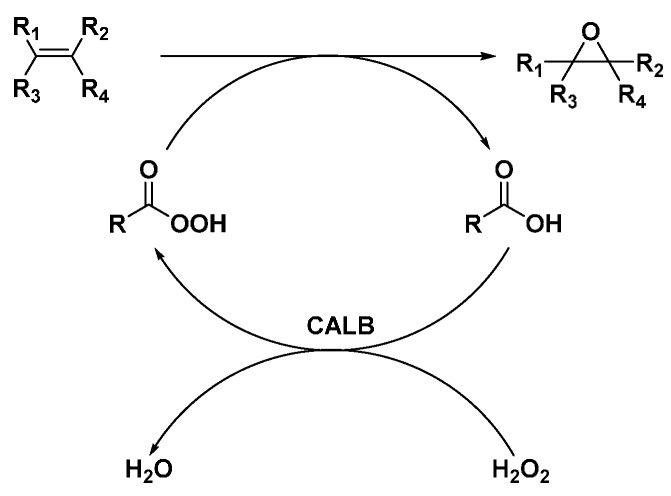
CALB catalyzed epoxidation of olefins in the presence of hydrogen peroxide.

It was reported that epoxidation of alkenes was achieved under extremely mild conditions by employing peroxycarboxylic acids formed continuously *in situ* by lipase-catalyzed peroxidation of the corresponding carboxylic acids as shown in Scheme 6.

Catalytic amount of octanoic acid was used to generate peroxiacid with H_2_O_2_ in hexane to epoxidize alkanes in hexane. CALB was found to be the most effective, but *Candida cylindracea*, *Humicola* and *Pseudomonas* also catalyzed the reaction. In contrast, *Mucor miehei* was found to be ineffective. 

## 3. Enzyme Catalysis in Polymer End-Functionalization

As mentioned before, our group pioneered quantitative polymer functionalization via CALB-catalyzed transesterification and Michael addition. Here we will briefly review this body of work for various polymers.

### 3.1. Poly(ethylene glycol) (PEG)

**Scheme 7:**
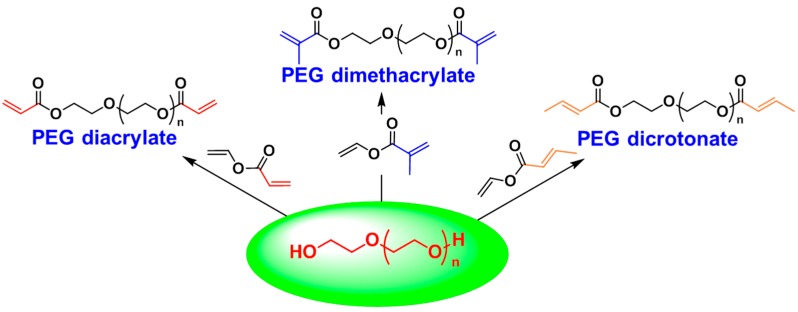
Functionalized PEGs vial CALB-catalyzed transesterification.

PEG is a non-toxic, hydrophilic polymer that is extensively used in medical science [41,42] including the applications of enhancing the circulation time and blood half-life for cell imaging, drug delivery, and antibody-based therapy [43,44,45]. However, the HO- end groups that are available for chemical derivatization are only a small fraction of the molecular mass of the polymer, and chemistries utilized for end-group modification must be high fidelity in nature and leave few or ideally no residuals [46,47]. Our group reported first the synthesis of quantitative end-functionalization of PEG catalyzed by CALB [48]. Functionalization of PEGs under solvent free conditions within 4 h was achieved by liquefying low molecular weight HO–PEG–OH (*M_n_* = 1050 and 2000 g/mol) at 50 °C and mixing it with the corresponding acyl donors (vinyl methacrylate, vinyl acrylate and vinyl crotonate) in the presence of immobilized CALB (Scheme 7). ^1^H- and ^13^C-NMR along with MALDI-TOF MS confirmed quantitative conversion with the expected structures [26,49,50]. 

**Scheme 8:**
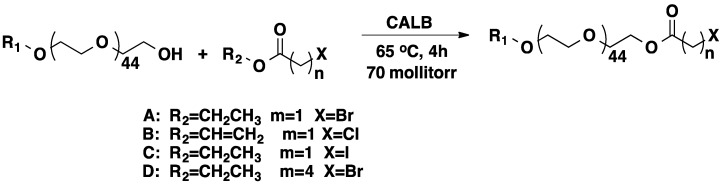
Enzymatic transesterification of halo-esters with PEGs in the presence of CALB.

Since halogens, especially bromine make a great leaving group, PEG-Br is often used as an intermediate for further functionalization. PEG is usually brominated using thionyl bromide or phosphorous tribromide in toluene [51,52]. An attractive alternative strategy is the use of enzymatic reactions. This “green” polymer chemistry approach offers many advantages, such as high efficiency, recyclability, and the ability to react under mild and solvent-free conditions [53]. Castano *et al.* showed that PEG can be halogenated (Scheme 8) in bulk when the polymer is heated with excess amount of the corresponding haloester at 65 °C for 4 h under vacuum (70 milliTorr) [53].

**Scheme 9:**
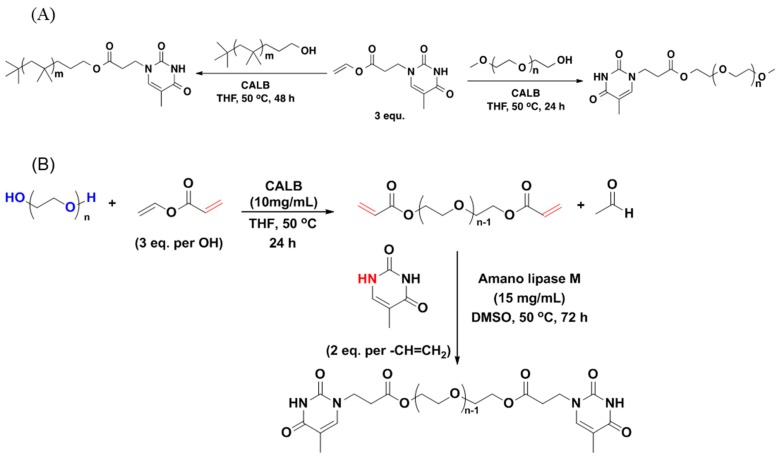
Enzyme [(**A**) CALB (**B**) *Amano* Lipase M] catalyzed thymine functionalization of polymer (PEG/PIB) [28].

Thymine functionalized PEG was synthesized through CALB catalyzed transesterification of the vinyl ester of thymine (Scheme 9A) [28]. 

In another process thymine-functionalized PEG was successfully prepared by the Amano lipase M-catalyzed Michael addition of thymine to PEG diacrylate within 72 h. The PEG-diacrylate was prepared by the transesterification of vinyl acrylate with HO-PEG-OH (*M_n_* = 2000 g/mol, *M_w_/M_w_* = 1.91) in the presence of CALB (Scheme 9B) [54].

### 3.2. Polyisobutylene (PIB)

PIB has commercial utility as a stabilizing fuel and motor oil additive, packaging elastomer, adhesive and sealant, and more recently, as a biomaterial. This saturated hydrocarbon elastomer has excellent thermal and oxidative stability, gas barrier properties, and biocompatibility [55]. Additionally there have been very successful materials developed that has harnessed the advantages and versatility of this polymer [55,56,57]. This is the case of poly(styrene-block-isobutylene-block-styrene) that has successfully been used as the drug-eluting coating on Boston Scientific’s TAXUS stents [57]. The same polymer is being investigated for ophthalmic implants to treat glaucoma, synthetic heart valves and other applications [56]. 

**Scheme 10:**
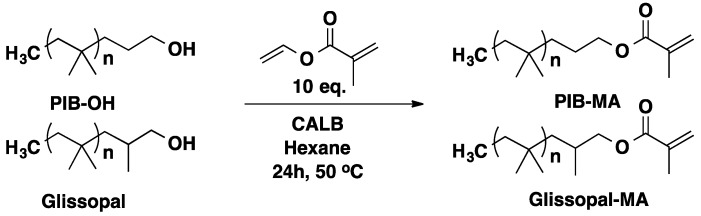
Enzymatic functionalization of PIB-OH and Glissopal-OH.

Quantitative methacrylation of hydroxyl-terminated PIB and Glissopal was achieved via CALB catalyzed transesterification of vinyl methacrylate at 50 °C in hexane in 24 h and without solvent in 2 h (Scheme 10) [49,54,58,59].

**Scheme 11:**
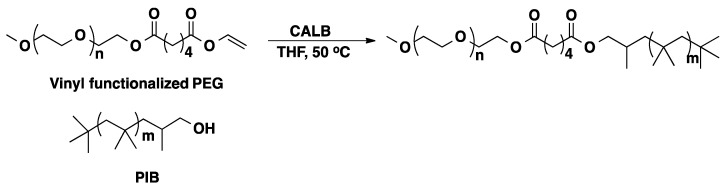
Preparation of poly(ethylene glycol)-b-polyisobutylene by enzymatic coupling [59].

Amphiphilic polymers, *i.e.*, products containing both hydrophilic and hydrophobic segments in the same molecule, are of interest for a variety of applications including cosmetic, pharmaceutical, detergent and biotechnological industries [60]. Diblock copolymers containing PIB and PEG were synthesized exploiting CALB catalyzed transesterification reaction. The copolymers were synthesized reacting vinyl functionalized PEG (synthesized by transesterification of divinyl adipate with PEG) with hydroxyl-functionalized PIB in THF at 50 °C in the presence of CALB (Scheme 11) [59]. 

**Scheme 12:**
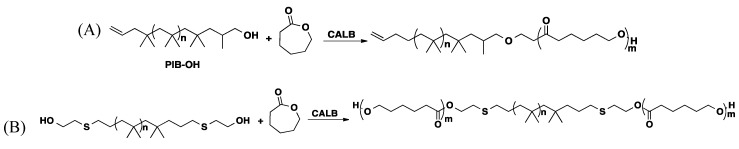
CALB mediated (**A**) bi- and (**B**) tri-block polymer synthesis.

Block copolymers of PIB with L-lactide and pivalolactone have been synthesized from *primary* hydroxyl functionalized PIBs and metal-containing activators. It was found that the blocks had phase-separated morphologies, and the crystallization behavior of the polylactide and polypivalolactone was influenced by the presence of the PIB blocks. Poly(ε-caprolactone-block-isobutylene-block-ε-caprolactone) block copolymers were synthesized using telechelic HO-PIB-OH macroinitiators obtained by multistep processes, and triethyl aluminum or HCl·Et_2_O catalyzed Ring Opening Polymerization (ROP). Our group found conditions leading to pure di- (Scheme 12A) and tri-block (Scheme 12B) copolymers—the details will be published elsewhere.

Thymine functionalized PIB were synthesized through CALB catalyzed transesterification reacting vinyl ester of thymine with hydroxyl functionalized PIB, similarly to that shown in Scheme 9A.

### 3.3. Polysiloxanes

**Figure 7:**
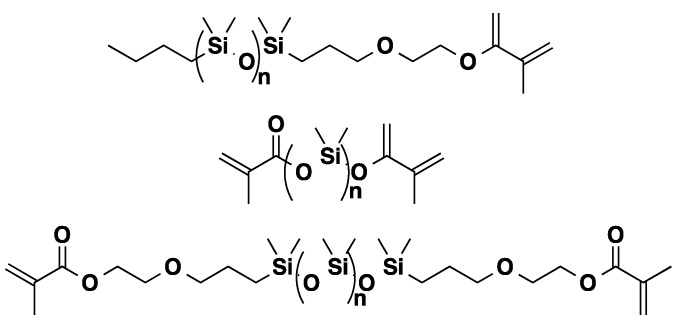
CALB catalyzed methacrylation of PDMS.

Commercially available polydimethylsiloxanes (PDMS) (*Mn* = 3200 g/mol), PDMS-monocarbinol (*Mn* = 5000 g/mol) and PDMS-dicarbinols (*Mn* = 4500 g/mol and 1000 g/mol) were also quantitatively methacrylated under solventless conditions within 2 h in the presence of CALB (Figure 7) [54]. 

### 3.4. Polystyrene

*Primary* hydroxy-functionalized polystyrene (PS-40(CH_3_)_2_Si-CH_2_-OH, M_n_ = 2600 g/mol; ĐM = 1.06) was quantitatively methacrylated by transesterification of vinyl methacrylate within 48 h.

### 3.5. Oligoesters

We explored the kinetics of CALB-catalyzed transesterification of divinyl adipate (DVA) with tetraethylene glycol (TEG). Like the mechanism shown in Figure 4, first, the nucleophilic serine (Ser105) residue interacts with one of the carbonyl groups of the DVA, forming a tetrahedral intermediate which is stabilized by the oxyanion hole of the enzyme via three hydrogen bonds, one from glutamine (Gln106) and two from threonine (Thr40) units. In the second step vinyl alcohol is released and tautomerized to acetaldehyde, and an acyl-enzyme complex is formed. In the third step, one of the HO– groups of TEG reacts to form a second tetrahedral intermediate, which is again stabilized by the oxyanion hole. In the last step, the enzyme is deacylated to form TEG- monovinyl adipate (V-TEG-OH in short form). Following this first cycle, the V-TEG-OH can react with an activated DVA, yielding difunctional V-TEG-V. The vinyl ester group of V-TEG-OH can also be activated by the enzyme. This complex then can react with another V-TEG-OH, yielding V-(TEG)_2_- OH or with HO-TEG-OH, yielding HO-(TEG)_2_-OH. Based on the kinetics conditions were optimized for the synthesis of symmetric and asymmetric telechelic oligoesters [21,26]. 

## 4. Regio- and Chemoselectivity in Enzyme-Catalyzed Polymer Functionalization

**Scheme 13:**
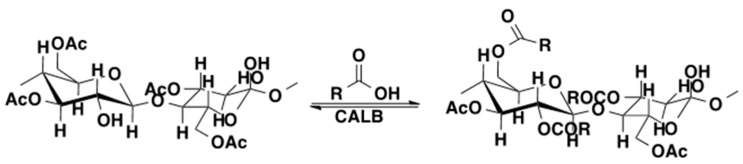
CALB-catalyzed acylation of cellulose acetate [61].

Enzyme-catalyzed reactions offer regio- and chemoselectivity in polymer functionalization as well. Site-specific chemical modification of hydroxy groups in polysaccharide chains is hardly possible via conventional organic synthetic method. Preparation of various esters was performed via CALB-catalyzed acylation of cellulose acetate [61] and hydroxypropyl cellulose [62] (Scheme 13). In the case of the acylation of cellulose acetate with lauric and oleic acids, the final conversion of both fatty acids was about 35% after 96 h of incubation at 50 °C. In the case of hydroxypropyl cellulose the final ester content was about 11% after 6-day incubation at 50 °C. Starch nanoparticles in microemulsions were reacted with vinyl stearate, ε-caprolactone, and maleic anhydride in the presence of CALB at 40 °C for 48 h to give starch esters with degrees of substitution (DS) of 0.8, 0.6, and 0.4, respectively. Substitution occurred regioselectively at the C-6 position of the glucose repeat units [63]. 

Another example is a novel regioselective strategy for the transesterification of *Konjac glucomannan* (KGM) with vinyl acetate using CALB in a solvent-free system. KGM is an abundant, naturally occurring polysaccharide isolated from the tubers of the *Amorphophallus konjac* plant. It consists of β-1,4-linked d-glucose and d-mannose units, and the molar ratio of glucose to mannose has been reported to be around 1 to 1.60. The Degree of Substitution (DS) depends on the temperature of reaction and varies from 0.34 to 0.58 at 30 °C and 60 °C respectively. It has also been found that the DS (from 0.67 to 0.16) of modified KGM sample decreases with increase in KGM molecular weight (from 114,000 to 980,000 g/mol) [64]. 

**Scheme 14:**
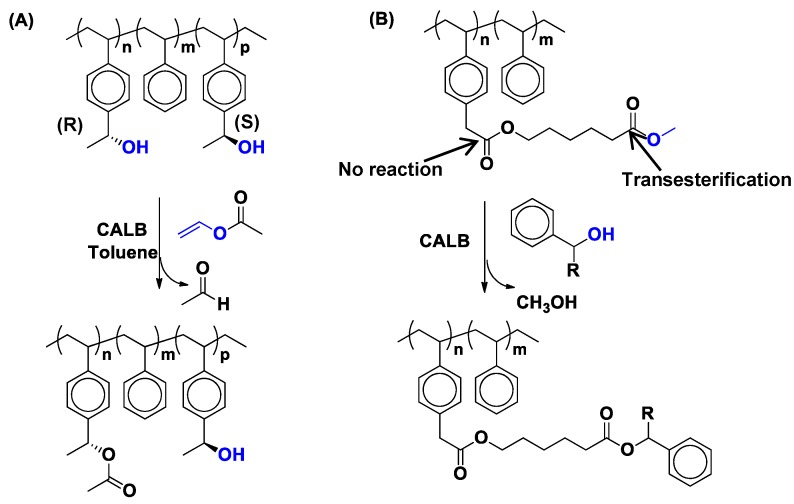
CALB-catalyzed transesterifications: (**A**) of vinyl acetate with the copolymer of styrene and 1-(4-vinylphenyl)ethan-1-ol (**B**) of the copolymer of styrene and methyl 6-(2-(4-vinylphenyl)acetoxy)hexanoate with benzyl alcohol (R = H) and 1-phenylethan-1-ol (R = -CH_3_).

Heise and coworkers reported the synthesis of chiral copolymers (with *M_n_* = 5000–6000 g/mol and *M_w_/M_n_* = 1.7–2.1) using styrene and 1-(4-vinylphenyl)ethan-1-ol, which contained about total 45% of the chiral monomer [1-(4-vinylphenyl)ethan-1-ol], [65]. Copolymers with the whole range of composition from 100% R to 100% S configuration were synthesized. It was found that CALB selectively catalyzed the transesterification reaction of the pendent alcohols with (R) configuration (Scheme 14A). 

75% of the pendent hydroxyl groups with (*R*) configuration were esterified in the presence of CALB over a time period of 24 h. Addition of excess ester and enzyme did not increase the conversion significantly. However, when copolymer containing pendent hydroxyl groups with 100% (*S*) configuration was subjected to transesterification reaction no desired grafted ester product was detected even after 48 h. In the case of copolymers containing pendent hydroxyl groups with a mixture of (*R*) and (*S*) configurations mostly the hydroxyl groups with *(R)* configuration were found to be esterified.

It was also observed that when copolymer of styrene and methyl-2-(4-vinylphenyl) acetate was reacted with benzyl alcohol, there was no reaction at all [66]. However, 78% conversion was obtained by introducing a spacer in the ester-functionalized polymer [methyl-6-(2-(4-vinylphenyl)acetoxy)-hexanoate] (Scheme 14B).

The -OH groups in the side chains of poly[*N*-(2-hydroxypropyl)-11-methacryloylaminoundecanamide-*co*-styrene], a comb-like polymer, and its corresponding monomer were acylated with vinyl acetate, phenyl acetate, 4-fluorophenyl acetate and phenyl stearate in THF in the presence of *Pseudomonas fluorescens*. The copolymer was acylated with about 40% conversion after 7 days when phenyl acetate was used as the acyl donor [67]. Poly(4-hydroxystyrene) was modified with aniline with *Polyphenol oxidase* catalyst, Only 1.3% of aniline was incorporated [68].

**Scheme 15:**
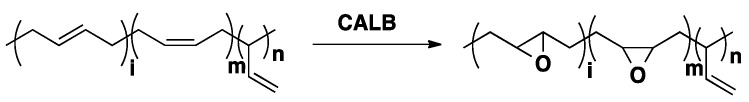
CALB-catalyzed epoxidation of polybutadiene.

Jarvie *et al.* showed that hydrogen peroxide and catalytic amount of acetic acid (10 wt %) can selectively epoxidize polybutadiene (*M_n_* = 1300 g/mol) (35% *trans*, 20% *cis*, 45% vinyl) in organic solvents in the presence of CALB (Scheme 15). The *cis* and *trans* alkene bonds of the polybutadiene backbone were epoxidized in yields of up to 60% while the pendant vinyl groups were untouched [69]. It has been reported earlier that CALB does not catalyzes the actual epoxidation reaction. However, it catalyzes the oxidation of the acid (acetic acid here) to generate peracid *in situ* in the presence of hydrogen peroxide which then traditionally reacts with the alkene resulting corresponding epoxidized material [70]. 

**Scheme 16:**
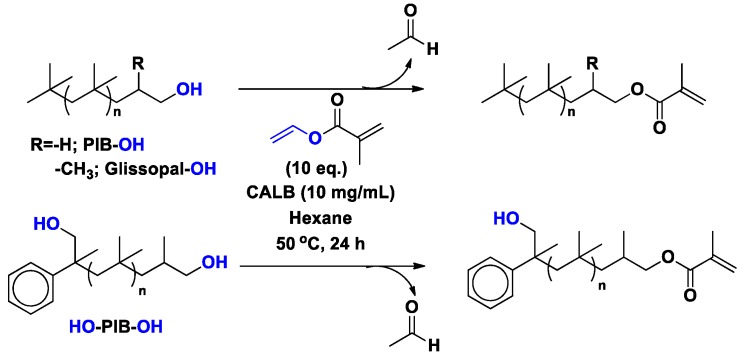
CALB-catalyzed methacrylation of PIB-OHs. PIB-OH (*M_n_* = 5200 g/mol, *M_w_/M_n_* = 1.09), Glissopal-OH (*M_n_* = 3600 g/mol, *M_w_/M_n_* = 1.34), and asymmetric telechelic HO-PIB-OH (*M_n_* = 7200 g/mol, *M_w_/M_n_* = 1.04).

Our group also reported regio- and stereospecificity in polymer functionalization. Specifically, asymmetric methacrylation of α,ω-hydroxy functionalized PIB was achieved by the regioselective transesterification of vinyl methacrylate using CALB in hexane within 24 h, leaving the sterically hindered hydroxyl group intact (Scheme 16). 

**Scheme 17:**
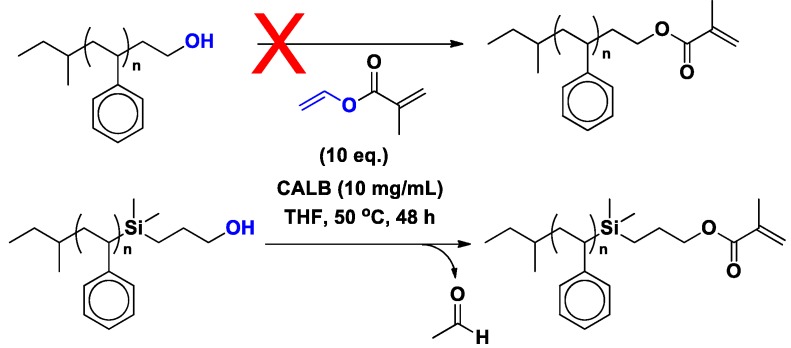
Unsuccessful transesterification of vinyl methacrylate with PS-OH prepared by ethylene oxide end-capping (*M_n_* = 2100 g/mol, *M_w_*/*M_n_* = 1.07) and quantitative transesterification of vinyl methacrylate with PS-OH prepared by end-capping with (CH_3_)_2_SiClH followed by hydroxylation with allyl alcohol (*M_n_* = 2600 g/mol, *M_w_*/*M_n_* = 1.06).

*Primary* hydroxy-functionalized polystyrene with a spacer (PS-(CH_3_)_2_Si-CH_2_-OH) [34,71] was quantitatively methacrylated by transesterification of vinyl methacrylate within 48 h. In contrast, the ethylene oxide end-capped PS-OH did not react (Scheme 17).

## 5. Limitations 

In terms of CALB-based transformations in solution: Challenges surround issues of incompatibility between the solvents tolerate by the CALB and the substrates solubility. In the case of solventless transformations, the substrate must be liquid at the working temperature (50 °C) and in the case of polycondensation the formation of highly viscous product limits the application. If the reaction proceeds for long periods of time, the CALB starts to catalyze the hydrolysis of the polyesters. As a result, it is still quite challenging to synthesize high molar mass polyesters. The control of polymerization in this case remains less efficient with enzymes than with chemical initiators.

## 6. Conclusions

Enzyme mediated functionalization of polymers can be used to generate new functionalized polymers. Chemical reactions specifically transesterification, Michael addition, ring opening polymerization can be conducted at milder conditions exploiting the catalytic activity of CALB. This method is green and can be used to synthesize polymers for biomedical applications as many cases no solvent and no metal catalysts were used. Enzymes also offer region- and chemoselectivity not achievable using conventional catalysis. 
